# Titanium Dioxide (TiO_2_) Nanoparticle Toxicity in a *Caenorhabditis elegans* Model

**DOI:** 10.3390/toxics11120989

**Published:** 2023-12-05

**Authors:** Sen-Ting Huang, Jian-He Lu, Sherwin M. Jualo, Lemmuel L. Tayo, Wan-Nurdiyana-Wan Mansor, Yi-Chieh Lai, Chih-Lung Wang, How-Ran Chao

**Affiliations:** 1 Department of Environmental Science and Engineering, National Pingtung University of Science and Technology, Pingtung County, Neipu 912, Taiwan; marssjason@gmail.com; 2 Department of Internal Medicine, Pingtung Veterans General Hospital, Pingtung County, Pingtung City 900, Taiwan; 3 Center for Agricultural, Forestry, Fishery, Livestock and Aquaculture Carbon Emission Inventory and Emerging Compounds, General Research Service Center, National Pingtung University of Science and Technology, Pingtung County, Neipu 912, Taiwan; toddherpuma@mail.npust.edu.tw; 4 School of Chemical, Biological and Materials Engineering and Science, Mapúa University, Intramuros, Manila 1002, Philippines; jualoshers@gmail.com (S.M.J.); lemueltayo@yahoo.ca (L.L.T.); 5 Faculty of Ocean Engineering Technology & Informatics, Universiti Malaysia Terengganu, Kuala Lumpur 21300, Malaysia; nurdiyana@umt.edu.my; 6 Department of Safety, Health and Environmental Engineering, National Kaohsiung University of Science and Technology, Yanchao, Kaohsiung City 824005, Taiwan; 7 Department of Civil Engineering and Geomatics, Cheng Shiu University, Niaosong District, Kaohsiung City 833, Taiwan; clwang@gcloud.csu.edu.tw; 8 Center for Environmental Toxin and Emerging-Contaminant Research, Cheng Shiu University, Niaosong District, Kaohsiung City 833, Taiwan; 9 Institute of Food Safety Management, College of Agriculture, National Pingtung University of Science and Technology, Pingtung County, Neipu 912, Taiwan; 10 School of Dentistry, College of Dental Medicine, Kaohsiung Medical University, Sanmin, Kaohsiung City 807, Taiwan; 11 Department of Occupational Safety and Health, Faculty of Public Health, Universitas Airlangga, Kampus C Mulyorejo, Surabaya 60115, Indonesia

**Keywords:** titanium dioxide, *Caenorhabditis elegans*, reproductive toxicity, neurobehavioral toxicity, toxic chemical

## Abstract

Titanium dioxide is a compound that is used in the food, cosmetic, and paint industries; however, it is still toxic to humans and the environment. This study determined the toxicities of titanium dioxide nanoparticles (TiO_2_ NPs) in a *Caenorhabditis elegans* (*C. elegans*) model. The effects of commercially available (C-TiO_2_) and synthetically (S-TiO_2_) prepared TiO_2_ NP solutions on lethality, lifespan, growth, reproduction, locomotion, and gene expression were studied in *C. elegans*. Exposure to TiO_2_ NPs (0.0, 0.01, 0.1, 1.0, and 10 mg/L) did not result in any change to the survival rate or body length of the nematodes, regardless of the concentration. However, there was a decrease in the reproduction (brood size) and locomotion (body bending and head thrashing) of the nematodes as the TiO_2_ NP concentration increased. The longevity of the nematodes was shortened following TiO_2_ NP exposure. The gene expression of sod-1, sod-3, ctl-1, ctl-2, cyp35A2, mlt-1, and mlt-2 in the nematodes showed that there was an overexpression of all genes when the worms were exposed to 1 mg/L C-TiO_2_ or 10 mg/L S-TiO_2_. It was therefore concluded that compared with S-TiO_2_, C-TiO_2_ possibly causes more toxicity or genotoxicity in the *C. elegans* model.

## 1. Introduction

Titanium dioxide (TiO_2_), also known as titanium (IV) oxide or titania, is a naturally occurring titanium oxide, occurring as a white, odorless, and non-flammable powder. It has attractive chemical and physical properties and is therefore widely used, for example, as a white pigment, because it is bright and has a high refractive index [1,2]. The global demand for TiO_2_ for the paint and coating industry in 2020 was approximately 3.6 million tons, accounting for 56% of the total mass production [3,4]. The engineered nanomaterials of TiO_2_ nanoparticles (TiO_2_ NPs) are also widely used in the paint, cosmetic, food, pharmaceutical, environmental treatment, thin film, and semiconductor industries and as photocatalysts [1]. TiO_2_ is also known as an E171 or INS171 food additive, which is a mixture of TiO_2_ defined as TiO_2_ NPs, due to its nano size allowing it to easily pass through the natural barriers of the human body into the lungs, liver, or digestive system [1]. As a food additive, TiO_2_ is E171 in the European Union (EU) and INS171 in the United States (USA) and was approved for use by the USA and EU in 1966 and 1969, respectively. The European Food Safety Authority (EFSA) stated that TiO_2_ NPs such as E171 are no longer considered safe, and the Food and Drug Administration in the USA (US FDA) accepted this agreement in 2021 [5]. E171 was banned by the EFSA in 2022. INS171 is continuously reviewed by the US FDA in terms of being restricted for use; hence, the safety of TiO_2_ NPs must be reviewed.

Although engineered nanomaterial TiO_2_ NPs are widely used within various industries, public and governmental concerns that the consumption of these compounds causes adverse health effects are increasing [6,7]. Toxicological studies regarding the effects of TiO_2_ NPs were conducted in various experimental models, suggesting that TiO_2_ NPs alter the cell cycle, cause apoptosis, affect the adhesion of the cell matrix, and induce DNA damage in human breast cancer cells [8,9]. An in vitro study of TiO_2_ NPs on the A549 cell line and co-cultured cells showed that there is a decrease in the viability of exposed cells and significant proinflammatory mediator (interleukin-8, IL-8) and cytokine (tumor necrosis factor-α, TNF-α) production increases for higher levels of TiO_2_ NPs [10]. The physicochemical properties of TiO_2_ NPs influenced the viability of A549 and co-cultured cells, but one study used TiO_2_ NPs to interfere with the epidermal growth factor receptor signaling pathway in human breast cancer cells to induce apoptosis [11]. TiO_2_ NPs are common, but they are potentially toxic; thus, it is crucial to gather information about the possible adverse effects of TiO_2_ NPs.

*Caenorhabditis elegans* (*C. elegans*) is a small, free-living soil nematode that is commonly used as a model organism for biological research because it is inexpensive, bioethical, and easily maintained [12]. It is used as an animal model for a wide range of toxicological studies, including studies of the toxic effects of fine particulate matter (PM_2_._5_) [13,14,15], nanoparticles such as graphene oxide, carbon nanotubes, and silica nanoparticles [16,17,18]. Studies of the toxic effects of TiO_2_ NPs on *C. elegans* have been previously conducted [19,20,21,22,23,24,25,26,27,28,29,30,31,32]. For most studies [19,20,21,22,23,24,25,26,27,28,29,30,31,32], the toxicological endpoints are the survival rate, locomotion, reproduction, lifespan, and induced oxidative stress for the nematodes. According to several reports, TiO_2_ NPs adversely affect the reproduction of *C. elegans*, and there was a constant decrease in the brood size as TiO_2_ NP concentrations increased [19,21,23,27]. Adverse effects on the nematodes were also induced by the crystallite particle size and crystalline forms of TiO_2_ NPs [20,24,28,29], along with phototoxicity to damage *C. elegans* [24]. Regarding neurological effects (e.g., locomotion), the frequency of head thrashing and body bending in the nematodes was shown to decrease when they were exposed to TiO_2_ NPs [21,26,27,31,32]. Additionally, several studies examined the interactions between TiO_2_ and toxic substances such as polystyrene particles, heavy metals, and pentachlorophenol, to evaluate their mixed toxicity when nematodes were subject to cotreatment [22,25,26].

This study determined the toxicity of TiO_2_ NPs using *C. elegans* as a model organism. Synthetically prepared (S-TiO_2_) and commercially available (C-TiO_2_) TiO_2_ NPs were used to treat the nematodes with different concentrations, and toxic effects were measured according to survivability, longevity, reproduction, locomotion, growth, and oxidative stress. The gene expression of TiO_2_-NP-exposed nematodes was utilized in an attempt to understand the reactive oxygen species (ROS)-induced activation in the present study.

## 2. Materials and Methods

### 2.1. Reagents and Raw Materials

*C. elegans* (Bristol strain N2) were purchased from the *Caenorhabditis* Genetics Center (CGC) (Minneapolis, MN, USA). Nematode growth medium (NGM) plates were produced using bacteriological agar, bactopeptone (Laboratories Conda, S.A., Madrid, Spain), and NaCl (Honeywell Fluka™, Morris, NJ, USA). Additional components for the plates include CaCl_2_, K_2_HPO_4_, and cholesterol from Sigma-Aldrich (St. Louis, MO, USA), and MgSO_4_ from Avantor Performance Materials, Ltd. (Gyeonggi-do, Republic of Korea). OP50 *Escherichia coli* (*E. coli*) from the Bioresources Collection and Research Center (Hsinchu, Taiwan) was used as food for the *C. elegans*, cultured using the Luria–Bertani broth from Sigma-Aldrich. The bleaching solution for the experiment was NaOCl from J.T. Baker (Central Valley, PA, USA) and KOH from Duksan Pure Chemicals (Gyeonggi-do, Republic of Korea). KH_2_PO_4_ from Avantor Performance Materials, LLC (Radnor, PA, USA), was used for the phosphate buffer, and Na_2_HPO_4_ from Honeywell Fluka™ was used for the M9 buffer. The K-medium was produced using NaCl from Honeywell Fluka™ and KCl from Avantor Performance Materials, Allentown, PA, USA. A dissecting microscope (Olympus, SZX10, Wlatham, MA, USA) was used for all physiological observations for the experiment.

### 2.2. Sample Collection and Preparation

S-TiO_2_ was produced using supercritical antisolvent precipitation, which is an environmentally friendly technology. The following is a summary of the precipitation procedure. Of 1M titanium isopropoxide (IV), 50 mL was dissolved in isopropanol and stirred in a high-pressure autoclave, with a gradual addition of 0.5 wt% surfactant dioctyl sulfosuccinate. CO_2_ was then pumped into the autoclave at 50 °C and 140 bar. The supercritical condition was maintained for 2 h, and CO_2_ was released via venting. The resulting powder was then maintained in a vacuum oven at 80 °C. C-TiO_2_ (Degussa P25, <25 nm particle size, 99.7% trace metals basis, Sigma-Aldrich) was also used for the experiments. The physical properties of C-TiO_2_ and S-TiO_2_ are shown in Table 1. The blown containers of S-TiO_2_ and C-TiO_2_ were covered with an aluminum foil stored at the electronic dry cabinet (D-82C, EDRY Co. Ltd, Taichung, Taiwan) in the dark. TiO_2_ NPs, including C-TiO_2_ and S-TiO_2_, were sonicated for 30 min at 40 kHz and 100 W for dispersion in the K-medium (50 mM NaCl, 30 mM KCl, 1.0 mg/L NaOAc, and PH of 6.0). A stock solution of 1.0 mg/mL was diluted to various concentrations with the K-medium before exposure. The individual exposure concentration (control, 0.01, 0.1, 1.0, and 10 mg/L) was then produced via the dilution of the stock solution.

### 2.3. Maintenance of C. elegans

Wild-type N2 *C. elegans* were maintained in NGM plates at 20 °C for incubation. OP50 *E. coli* were incubated as a food source in the Luria–Bertani broth overnight at 37 °C. Age-synchronized nematodes were cultured using alkaline bleaching. Plates containing a high density of eggs and gravid nematodes were re-washed with ddH_2_O and then placed in a centrifuge tube. The gravid nematodes were then lysed using the bleaching solution to obtain the eggs. The eggs were maintained in a Petri dish containing an M9 buffer and were incubated at 20 °C for 12–48 h to produce age-synchronized L1 worms used for the experiments.

### 2.4. Acute Exposure

The experiments utilizing the *C. elegans* model in the present study are listed in Table 2. The nematodes (*C. elegans*) were used for acute exposure according to the standard methods, with minor changes [33,34]. The age-synchronized L1 worms underwent incubation until the L3 or early L4 stages. Then, the worms were gently washed from the plate using the K-medium and centrifuged at 2500× *g*. The *E. coli* residues were removed via the aspiration of the supernatant, without disturbing the worm pellet. The pellet was then re-suspended via the continuous pipetting of the K-medium into the pellet. The seeding volume was calculated via pipetting two drops onto a glass slide and then manually counting the worms. Approximately 200 worms were seeded per well in a 12-well plate, where each well contained 1 mL of the different exposure concentrations for each sample, diluted with the K-medium. The wells were then incubated at 22 °C for 24 h without food. The synchronized nematodes were exposed to C-TiO_2_ or S-TiO_2_ in the K-medium in a dark environment to prevent ultraviolet (UV) radiation as much as possible. The 12-well plates, NGM plates, and 6 cm petri dishes containing the nematodes immersed in the K-medium were wrapped with an aluminum foil to maintain the dark conditions.

### 2.5. Lethality Assay

Fifty exposed nematodes of *C. elegans* were transferred to a fresh NGM plate without OP50. The nematodes were then prodded using a worm picker to assess whether they were dead or alive. Nematodes that were non-responsive to the stimulation were deemed dead. The assay was performed in triplicate for each exposure concentration.

### 2.6. Lifespan Assay

Fifty exposed nematodes (the untreated control, 0.01, 0.1, 1, and 10 mg/L) were transferred to a NGM plate containing food (OP50). The nematodes were then transferred to a fresh NGM plate every day for the first 4–5 days until the egg-laying period. After the egg-laying period, the nematodes remained in place and were incubated at 22 °C. The nematodes were assessed daily for 24 days, which is the general lifespan of *C. elegans*, and were scored as alive, censored (lost), or dead using a worm picker to softly provoke them. Three lifespan assays were conducted for each exposure concentration. The mean lifespan was calculated employing the method described in the previous reports [13,16].

### 2.7. Reproductive Assay (Brood Size Calculation)

When the nematodes were exposed to the different concentrations of TiO_2_ NPs (the untreated control, 0.01, 0.1, 1.0, and 10 mg/L), 50 age-synchronized worms were transferred daily to a fresh NGM plate containing OP50 during the first 4–5 days of the egg-laying period at 22 °C. An L4-exposed nematode was placed in each well of a 12-well NGM plate containing OP50 to calculate the brood size. The nematodes were transferred to a new plate every 2 days during the egg-laying period. The old plates containing eggs were incubated, and the eggs were allowed to grow until the L4 stage so the number of progeny could be counted. The total progeny for each worm was recorded, and a total of 30 nematodes were used for each exposure concentration.

### 2.8. Locomotion Assay (Head Thrashing and Body Bending)

Locomotion behavior, such as head thrashing and body bending in the *C. elegans* model, is shown as the neurological function of the motor neuron, as described previously [13,16]. Head thrashing is defined as a change in the direction of the midbody of the nematode, and body bending is defined as a change in the direction of the posterior bulb of the pharynx of the nematode along the *y*-axis, assuming it is traveling along the *x*-axis. The locomotion of each nematode was observed using a dissecting microscope (Olympus, SZX10, Waltham, MA, USA). Head thrashing was measured for 1 min, and body bending was measured for 20 s. Thirty nematodes were used, and experiments were performed in triplicate for each exposure concentration (control and 0.01, 0.1, 1.0, and 10 mg/L).

### 2.9. Growth Measurement Assay

L1 young nematodes were exposed to different concentrations of TiO_2_ NPs for a day and transferred to NGM plates with OP50 to be incubated until growth to the L4 stage or young adult stage. The nematodes were maintained at a high temperature of 66 °C in a dry bath for 5 min to straighten their body and allow for easier body length measurement. A dissecting microscope was used to capture images and ImageJ (http://imagej.nih.gov/ij/, accessed on 12 October 2023) (Madison, WI, USA), which is an open-source Java-based image software, was used to measure the length of each nematode.

### 2.10. Gene Expression Assays (Quantitative Real-Time PCR Assays)

L3/young L4 nematodes of 500–1000 worms were dispersed in 6 cm Petri dishes after exposure to different TiO_2_ NP concentrations (untreated control, 0.01, 0.1, 1, and 10 mg/L). The nematodes were gathered, and total RNA was extracted from each exposed sample using the TRIzol Reagent (Gibco, Life Technologies, Carlsbad, CA, USA).

To test for cDNA synthesis, 1000 ng of RNA was reversed to cDNA using a High-Capacity cDNA Reverse Transcription kit (Gibco, Life Technologies, Carlsbad, CA, USA). The real-time PCR was analyzed using the following processes. cDNA of 50 ng was amplified using the SYBR green PCR master mix (Gibco, Life Technologies, Carlsbad, CA, USA) for a 10 min 95 °C denaturation stage, 40 15 sec repetitions at 95 °C, and finally 1 min at 60 °C using an Applied Biosystems PRISM 7500 fast real-time PCR system. To measure the gene expression of the nematodes, quantitative methods were used to measure the expression of superoxide dismutase-1 (sod-1), sod-3, cyp-35A2, catalase-1 (ctl-1), ctl-2, melatonin-1 (mtl-1), mtl-2, and actin-1 mRNA. The sequences for the primers are shown in Table 3.

### 2.11. Statistical Analysis

Measurements of the brood size, locomotion, length, lethality, and gene expression of *C. elegans* did not produce a normal distribution for the Shapiro–Wilk tests, so nonparametric tests were used for other statistics. For the survival rates, a survival plot or the Kaplan–Meier plot was used to calculate the effect of TiO_2_ NPs on the lifespan of the nematodes. A Kaplan–Meier plot was constructed using GraphPad Prism 6 (San Diego, CA, USA). The significance of each day for the testing was determined, and the Kruskal–Wallis *H* test was used to select days for comparison to the control using the Mann–Whitney *U* test. All statistical analyses for the experiments used SPSS version 12 (International Business Machines Corp., New York, NY, USA).

## 3. Results

### 3.1. Lethality and Growth Measurements

**Figure 1:**
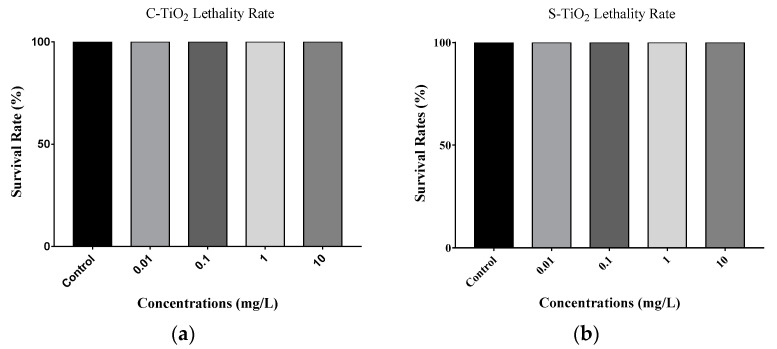

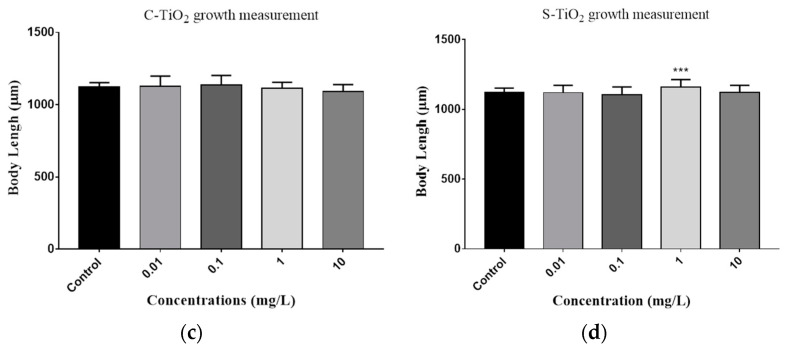
Nano-toxic effect of TiO_2_ on the survival rate and body length of the *C. elegans* models: (**a**) toxicity on survival rates after *C. elegans* was exposed to commercially available TiO_2_ (C-TiO_2_); (**b**) toxicity on survival rates after *C. elegans* was exposed to synthetically produced TiO_2_ (S-TiO_2_); (**c**) toxicity on body length after *C. elegans* was exposed to C-TiO_2_; and (**d**) toxicity on body length after *C. elegans* was exposed to S-TiO_2_. *** *p* < 0.001.

Figure 1a,b show the survival rates for *C. elegans* when the nematodes were exposed to TiO_2_ NPs, including C-TiO_2_ and S-TiO_2_. The results show that there are no significant differences between the survival rates for exposed nematodes and the control groups after exposure to C-TiO_2_ and S-TiO_2_. The growth of the untreated controls and the exposed nematodes is shown in Figure 1c for C-TiO_2_-treated worms and Figure 1d for S-TiO_2_-treated worms. When nematodes were exposed to C-TiO_2_ and S-TiO_2_, there was no significant change in body length for any of the concentrations, including the controls.

### 3.2. Reproduction and Locomotion

**Figure 2:**
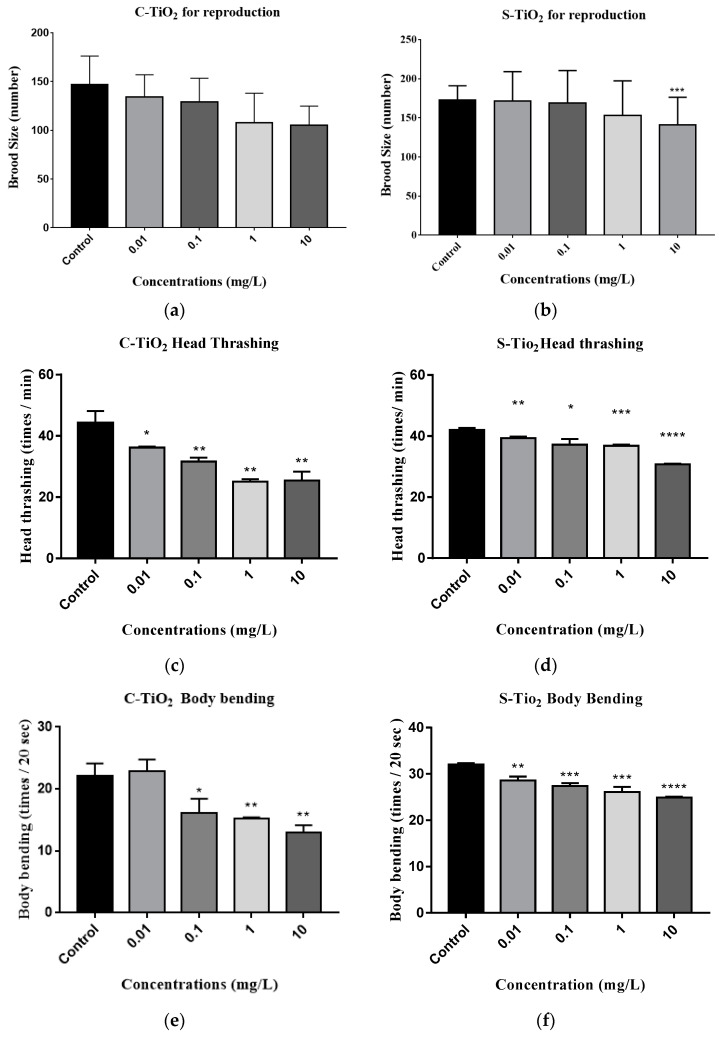
Nano-toxic effects of TiO_2_ on the reproduction and locomotion behavior of the *C. elegans* models: (**a**) reproductive toxicity after *C. elegans* was exposed to C-TiO_2_; (**b**) reproductive toxicity after *C. elegans* was exposed to S-TiO_2_; (**c**) locomotion in terms of head thrashing when nematodes were exposed to C-TiO_2_; (**d**) locomotion in terms of head thrashing when nematodes were exposed to S-TiO_2_; (**e**) locomotion in terms of body bending when *C. elegans* was exposed to C-TiO_2_; and (**f**) locomotion in terms of body bending when *C. elegans* was exposed to S-TiO_2_. The significant differences are expressed as * *p* < 0.05, ** *p* < 0.01, *** *p* < 0.001 and **** *p* < 0.001 between the control and the exposed groups.

Figure 2a,b show the effect of exposure to C-TiO_2_ or S-TiO_2_ on the reproduction of *C. elegans*, and Figure 2a shows the change in brood size (reproduction) for the nematodes exposed to C-TiO_2_ in concentrations from 0.01 to 10 mg/L. The data show a significant decrease in brood size of 2.3, 11.3, and 18.5% for concentrations of 0.1 (*p* = 0.009), 1.0 (*p* < 0.001), and 10 (*p* < 0.001) mg/L, respectively, compared to the unexposed control group. Figure 2b shows the change in brood sizes when nematodes are exposed to S-TiO_2_. There is a significant decrease of 28.0% (*p* < 0.001) for a dose of 10 mg/L compared to the control group.

The neurological effects on locomotion for the *C. elegans* that were exposed to TiO_2_ NPs are shown in Figure 2c–f. For nematodes that were exposed to C-TiO_2_, the frequencies of head thrashing and body bending for different doses are shown in Figure 2c,e. Compared with the controls, there was a significant reduction of 18.1, 28.6, 43.3, and 42.6% for doses of 0.01 mg/L (*p* = 0.011), 0.1 (*p* < 0.001), 1.0 (*p* < 0.001), and 10 mg/L (*p* < 0.001), respectively, for head thrashing and 27.6, 31.0, and 41.4% for doses of 0.1 (*p* = 0.035), 1.0 (*p* < 0.001), and 10 (*p* < 0.001) mg/L, respectively, for body bending in worms that were exposed to C-TiO_2_.

For nematodes that were exposed to S-TiO_2_, there was a decrease in head thrashing and body bending as the S-TiO_2_ concentration increased (Figure 2d,f). For nematodes that were exposed to S-TiO_2_, there was a significant decrease in the frequency of head thrashing for concentrations of 0.1 mg/L (*p* = 0.008), 1.0 (*p* = 0.021), and 10 mg/L (*p* < 0.001), resulting in decreases of 11.5, 12.4, and 27%, respectively, in comparison with the control. Figure 2f shows that the frequency of body bending for nematodes exposed to S-TiO_2_ was significantly less than that of the untreated control nematodes; there was a reduction of 10.7, 14.4, 18.5, and 22.2% at doses of 0.01 (*p* = 0.009), 0.1 (*p* < 0.001), 1.0 (*p* < 0.001), and 10 mg/L (*p* < 0.001), respectively. The results show that nematodes exposed to S-TiO_2_ were more significantly negatively affected in terms of reproduction and locomotion than nematodes exposed to C-TiO_2_.

### 3.3. Lifespan

**Figure 3:**
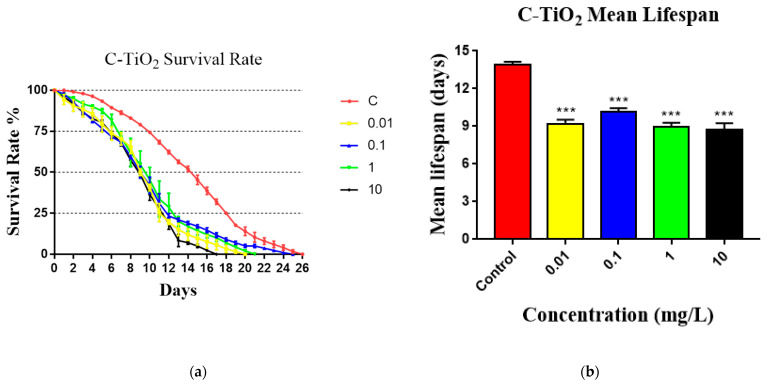

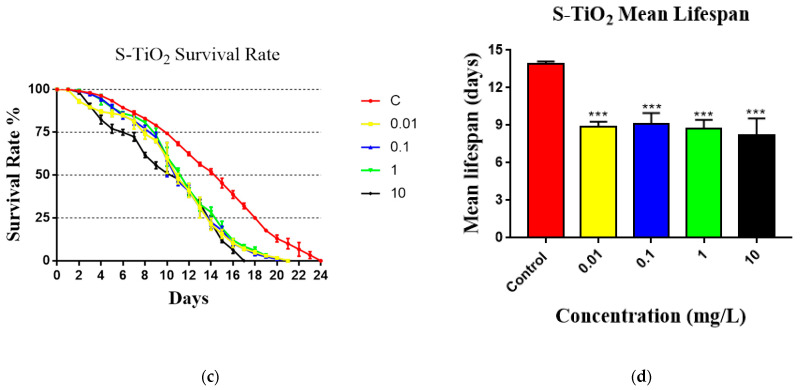
The lifespan of *C. elegans* that were exposed to various TiO_2_ concentrations (0, 0.01, 0.1, 1, 10 mg/L) is shown as: (**a**) survival rate for *C. elegans* after exposure to commercial TiO_2_ (C-TiO_2_); (**b**) average lifespans (mean lifespans) of nematodes after exposure to various concentrations of C-TiO_2_; (**c**) survival rate for *C. elegans* after exposure to synthesized TiO_2_ (S-TiO_2_); and (**d**) average lifespan of nematodes after exposure to various concentrations of S-TiO_2_. The significant differences are expressed as *** *p* < 0.001 between the control and the exposed groups.

The lifespan of *C. elegans* is shown in Figure 3. Nematodes were exposed to 0, 0.01, 0.1, 1.0, and 10 mg/L of C-TiO_2_ or S-TiO_2_ NPs. Compared with the controls, the lifespan curves of *C. elegans* were obviously lower when the nematodes were exposed to C-TiO_2_ or S-TiO_2_ (Figure 3a,c). Figure 3a shows that the longevity of the control nematodes was notably higher than those of the exposed groups in all the C-TiO_2_-treated worms from the first day to the final day. For S-TiO_2_-treated worms (Figure 3c), the survival curves were not significantly different in the first 8 days between the control and the 0.01–1.0 mg/L concentration groups, but significantly different longevity is evident between the control and the 0.01–1.0 mg/L concentration groups from the 10th day. The longevity of the 10 mg/L S-TiO_2_-treated worms was always significantly and notably lower than that of the control from the first few days to the final day. There was a significantly higher mean lifespan of the control in comparison to the exposed groups for the toxic substances of C-TiO_2_ and S-TiO_2_ (Figure 3b,d). The mean lifespan of C-TiO_2_-exposed worms was significantly shorter compared with the untreated control. Similarly, our study indicates that S-TiO_2_-exposed nematodes have reduced longevity.

### 3.4. Gene Expression

**Figure 4:**
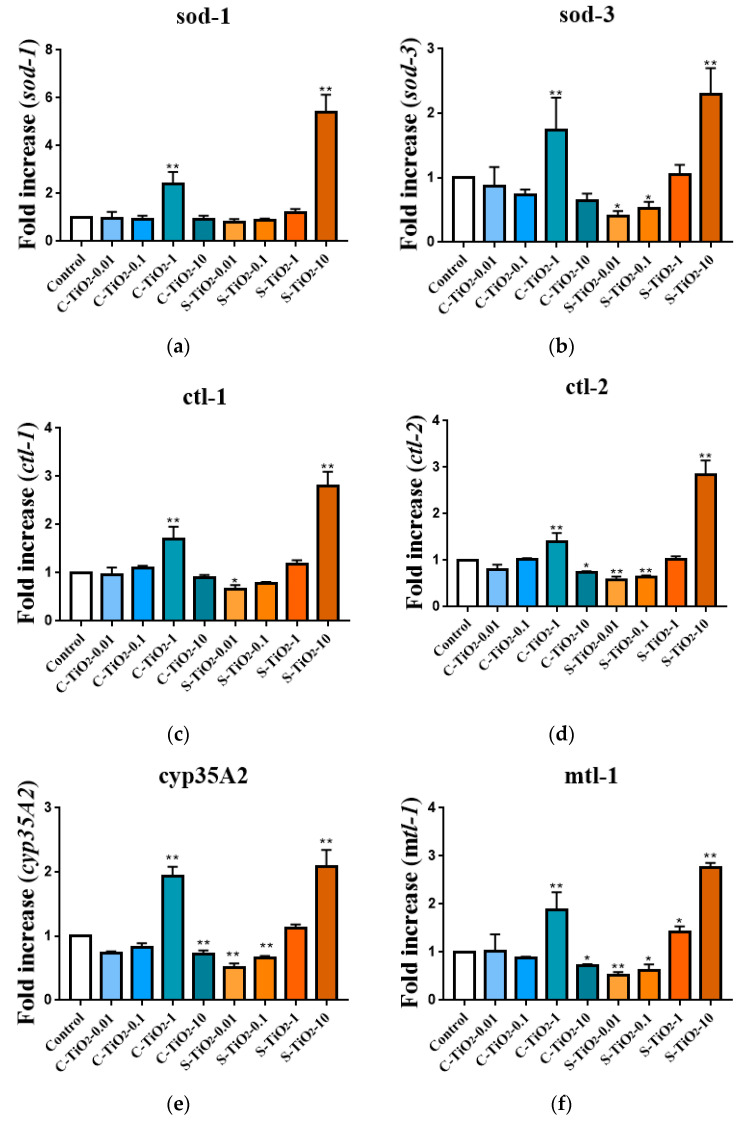

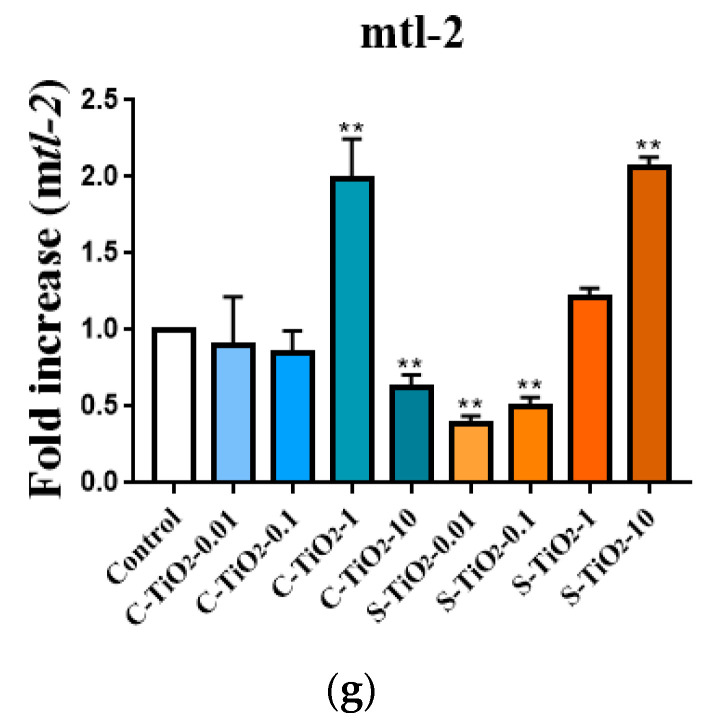
Gene expression of (**a**) sod-1, (**b**) sod-3, (**c**) ctl-1, (**d**) ctl-2, (**e**)cyp35A2, (**f**) mlt-1, and (**g**) mlt-2 in *C. elegans* that were exposed to various commercial or synthesized TiO_2_ (C-TiO_2_ or S-TiO_2_). C-TiO_2_-0.01 signifies 0.01 mg/L of C-TiO_2_. The increases are compared with untreated controls. The significant differences are expressed as * *p* < 0.05 and ** *p* < 0.01 between the control and the exposed groups.

For the *C. elegans* models in this study, sod-1 and sod-3 encoding sod and ctl-1, and ctl-2-encoding catalases were used to determine oxidative stress. Stress response was regulated by mlt-1- and mlt-2-encoding metallothioneins genes in this study. Oxidative stress is induced when there is an accumulation of ROS in the nematode. In *C. elegans*, the genes superoxide dismutase (1–5) and catalase (1–3) play a role in the antioxidant and stress response for nematodes [22,35]. The genes of mlt-1 and mlt-2 play a primary role in the detoxification of heavy metals in nematodes, and the cyp35a2 gene is involved in the metabolism of endogenous compounds, xenobiotic activity, and oxidation [36,37]. This study investigated the gene expression of sod-1, sod-3, clt-1, clt-2, cyp35A2, mlt-1, and mlt-2 in *C. elegans* after exposure to C-TiO_2_ or S-TiO_2_. The U sharp for dose-responsive curves for nematodes that were exposed to C-TiO_2_ or S-TiO_2_ is shown in Figure 4. For worms exposed to C-TiO_2_ at 1.0 mg/L C-TiO_2_, there was a 141, 74.3, 70.3, 39.3, 93.5, 88.4, and 98.7% significant increase in sod-1, sod-3, clt-1, clt-2, cyp35A2, mtl-1, and mtl-2 genes, respectively, compared with the controls. There was a negative activation on C-TiO_2_ at the doses of 10 mg/L for gene expression. There was a significant reduction of 26.8, 20.3, 36.9, and 27.5% for the clt-2, mtl-1, mtl-2, and cyp35A2 genes, respectively, and the doses of 0.01 and 0.1 mg/L produced a significant reduction of 26.9 and 18.3% for the cyp35A2 gene, respectively, in *C. elegans* compared with the controls.

The U sharp responses were determined for the genotoxicity of the *C. elegans* that were exposed to S-TiO_2_. There was a positive change in gene expression when nematodes were exposed to 1.0 and 10 mg/L S-TiO_2_. There was a significant increase for the doses of 10 mg/L S-TiO_2_ (sod-1: 440%; sod-3: 130%; clt-1: 180%; clt-2: 185%; cyp35A2: 108%; mtl-1: 177%; and mtl-2: 106%) and 1 mg/L S-TiO_2_ (mtl-1: 41.0%). There was a negative change in the gene expression of *C. elegans* that were exposed to S-TiO_2_ at the doses of 0.01 and 0.1 mg/L. There was a decrease in the gene expression for sod-3 (0.01 and 0.1 mg/L: 60.0% and 47.9%, respectively), clt-1 (0.01 mg/L: 33.9%), clt-2 (0.01 and 0.1 mg/L: 42.8% and 36.3%, respectively), cyp35A2 (0.01 and 0.1 mg/L: 49.3% and 34.4%, respectively), mtl-1 (0.01 and 0.1 mg/L: 47.6% and 38.4%, respectively), and mtl-2 (0.01 and 0.1 mg/L: 61.3% and 50.3%, respectively), as shown in Figure 4.

## 4. Discussion

In the past, TiO_2_ was recognized as a low-toxicity food additive [38]. More recently, several reports have assessed the safety of food-grade TiO_2_ to provide updated evidence that EFSA is no longer considered safe due to its high composition of NPs [5,39,40]. This study shows that an acute exposure to either C-TiO_2_ or S-TiO_2_ has no significant effects on the survival rate or the body length of *C. elegans* for any concentration of TiO_2_ NPs. These results are consistent with those of previous studies reporting on the effects of TiO_2_ NPs on *C. elegans*, which indicated that there was no significant effect on the survival rate or body length of the nematodes [28,32]. A similar in vivo study reported a significant decrease in the survival rate and body length of nematodes for *C. elegans* after prolonged exposure to TiO_2_ NPs at 50 µg/L (0.05 mg/L), but exposure to lower concentrations (from 0.05 µg/L to 10 µg/L) produced no significant effect [41]. Two previous studies determining the effect of TiO_2_ on *C. elegans* under soil conditions showed that there was a decrease in the survival rate and body length of nematodes as the concentration of TiO_2_ increased [26,31]. The results of the present study for body length and survival are not in agreement with those of some studies involving (i.e., soil) exposure to TiO_2_ NPs over a long period or within the environment. For the nematodes’ longevity, there were significant differences in lifespans between the controls and TiO_2_ NP-exposed groups. Ma et al. (2019) indicated that the longevity of the *C. elegans* in the exposed groups was shortened after the nematodes were acutely exposed to TiO_2_ NPs (Degussa P25) of 1.0 (mean lifespan = 12.6 days) and 10 (mean lifespan = 13.3 days) mg/L compared with the control (mean lifespan = 17.3 days) [24]. The results of the present study are similar to those of Ma’s study regarding exposure pattern and the same doses.

Regarding the reproductive effect of the nematodes, there was a significant decrease in the brood size of the nematodes exposed to TiO_2_ NPs, including C-TiO_2_ and S-TiO_2_. Previous studies also reported a negative correlation between the concentrations and reproduction for worms exposed to TiO_2_ NPs [21,22,24,30,32,41]. A previous study that determined the effect of the anatase-phase TiO_2_ NPs and rutile-phase TiO_2_ NPs showed that there was a concentration-dependent decrease in reproduction after exposure to 500 µg/mL anatase phase TiO_2_, and the nematode population decreases by 52% [32]. Another study showed that the primary particle size and the crystalline structure had a significant effect on the toxicity of TiO_2_ NPs [29]. The results for this study also show that C-TiO_2_, which has larger pores and crystallites than S-TiO_2_, had a much more significant effect on the reproduction of *C. elegans*. For the neurological effects, nematodes that were exposed to C-TiO_2_ showed a significant decrease in head thrashing and body bending compared to those that were exposed to S-TiO_2,_ particularly for high doses. The results for locomotion were similar to those for the effect on reproduction. As the TiO_2_ NP concentration increased, there was a decrease in head thrashing and body bending. These results are consistent with those of previous studies [13,23,31,32,35,42]. Wu et al. (2013) showed that head thrashing and body bending decreased as the concentration of TiO_2_ increased [41]. Wu’s study showed that an acute exposure of nematodes resulted in a concentration-dependent decrease in locomotion [42]. Previous studies showed that the exposure of nematodes to TiO_2_ NPs affected nine metabolic pathways in *C. elegans*, with a notable emphasis on the tricarboxylic acid (TCA) cycle, which exhibited the most significant change after exposure to TiO_2_ [20]. Within this cycle, glutamate plays a central role as a key metabolite for the synthesis of γ-aminobutyric acid (GABA). GABA acts predominantly at neuromuscular synapses in *C. elegans*, directly affecting the locomotion of nematodes [43]. There was a decrease in GABA expression after exposure to TiO_2_ NPs, and this was probably the underlying cause of the significant decrease in the nematodes’ locomotive and reproductive functions.

The induced toxicity via TiO_2_ was inked to their particle size, whereby bulk TiO_2_ (not nano-type) caused more toxic effects, including lethality, locomotion, reproduction, longevity, toxic genes, and metabolomics, than TiO_2_ NPs [20,24,28,44]. However, compared with the larger sizes of TiO_2_ NPs (60 and 90 nm) at a starting concentration of 10 mg/L, Wu’s study used nematodes treated with smaller TiO_2_ NPs (4 and 10 nm) to note a more significant decrease in locomotion from 1.0 to 50 mg/L TiO_2_ NPs [42]. The crystallite particle sizes of C-TiO_2_ and S-TiO_2_ were 19.2 and 9.5 nm, respectively, and the tests for body bending and head thrashing showed a significant reduction at 1.0 mg/L in the present study. Wu’s study [42] also showed that small TiO_2_ NPs (e.g., 4 and 10 nm) caused a more harmful effect than large particles of TiO_2_ NPs (60 and 90 nm), particularly in terms of head thrashing, body bending, body length, and lethality. The present study and Wu’s report support the hypothesis that particle size is an important factor in TiO_2_ NP toxicity.

Several studies demonstrated that the different crystal forms of TiO_2_ NPs (e.g., anatase and rutile) induce different levels of toxic effects, including lethality, physiological traits, locomotion, reproduction, lifespan, and gene expression, in *C. elegans* [20,28,31,32]. According to the patterns obtained via X-ray diffraction (XRD) in C-TiO_2_ (Degussa P25), which is a mixture of 80–86% anatase and 14–20% rutile, and S-TiO_2_, which is almost pure anatase (>99%) that is made in an environmentally friendly manner in a laboratory, the XRD images were similar because these substances are anatase-rich. Hu et al. (2018) showed more harmful neurological effects on rutile-treated nematodes than on anatase-treated ones [32]. C-TiO_2_ NPs induced more toxic effects than S-TiO_2_ NPs, probably due to the 16–20% rutile contents in C-TiO_2_ NPs in the present study. A good explanation for this may be that the major factor in the different toxicity between C-TiO_2_ and S-TiO_2_ is related to the crystal structure, and the crystallite particle size is a minor contributor.

This study determined the effects of C-TiO_2_ and S-TiO_2_ on the gene expression of sod-1, sod-3, ctl-1, ctl-2, cyp35a2, mlt-1, and mlt-2 in *C. elegans*. There was an overexpression of all the genes in the nematodes after exposure to 1 mg/L of C-TiO_2_. There was also an overexpression of all genes after exposure to 10 mg/L S-TiO_2_, but this increase for S-TiO_2_ was inconsistent with the inverse trend for C-TiO_2_ dosages of more than 1 mg/L. For S-TiO_2_, there was significant overexpression of all genes at 10 mg/L, and sod-1 was the most significantly overexpressed, with a 440% increase compared to the control. Compared with worms exposed to S-TiO_2_, the experiments showed that the worms exposed to C-TiO_2_ generated more genotoxic effects or ROS-induced toxicity. A previous study of the effect of TiO_2_ NPs on the expression of different ROS in *C. elegans* showed that there was a slight decrease in the expression of sod-1 and a significant increase in the expression of sod-3 [22]. The results of this study may be different because the previous study used prolonged exposure and a lower concentration of TiO_2_, at 1 µg/L.

The nematodes with mutated ROS-related genes, including sod-2, sod-3, mtl-2, and hsp-16.48, rendered nematodes more susceptible to the toxicity of TiO_2_ NPs [27]. Prof. Wang and his team members showed that nematodes that were exposed to TiO_2_ NPs at concentrations of 1.0 to 100 mg/L induced ROS production [31,43]. If *C. elegans* were chronically exposed to low doses of 1.0, 10, and 100 μg/L, then there was a significant production of ROS only for small particles of 4 and 10 nm [43]. Wu’s study [31] showed that ROS activation was inversely correlated with locomotion behavior, including body bending and head thrashing. An increase in the expression of these ROS genes signified oxidative stress within the nematodes, and the activation of these genes is a component of nematodes’ antioxidant defense system. Excessive production of these free radicals can overpower the defense mechanism, resulting in oxidative stress. Furthermore, this oxidative stress can induce cellular damage and disturb the metabolic pathways, producing a toxic effect in nematodes. Previous studies used genomic mutants of *C. elegans* and these caused adverse effects via the expression of specific genes [22,24,28]. When *C. elegans* were exposed to 25 mg/L TiO_2_ NPs, there was a more significant decrease in reproductive function (brood size) and locomotion behavior (body bending and head thrashing) in the sod-2, sod-3, mtl-2, and hsp-16.48 mutants than in the normal wild-type strain [27]. Rui et al. (2013) [27] also studied ROS production to show that these four mutant stains (sod-2, sod-3, mtl-2, and hsp-16.48) produced a greater decrease in ROS production than the normal wild-type stain at 25 mg/L TiO_2_ NPs.

This study was limited in several ways. First, our goal was to examine nano-toxicity induced by TiO_2_ NPs in a food additive. The dosages (0.01, 0.1, 1.0, and 10 mg/L) of TiO_2_ NPs used in this study covered the spectrum relevant to the potential exposure levels in food products. The doses of S-TiO_2_ and C-TiO_2_ were not considered or referenced from the environmental levels. Second, C-TiO_2_ was purchased from a commercial company and S-TiO_2_ was produced using environmentally friendly technology in the laboratory to increase the purity. According to the data obtained using XRD, S-TiO_2_ and C-TiO_2_ presented similar crystal structures of anatase-rich crystallites. It was very difficult to examine TiO_2_ toxicity based on the different crystallite forms in our study. Third, the anatase-rich crystallites of C-TiO_2_ and S-TiO_2_ easily induced high-quality photocatalytic activities to generate photoexcited electrons and positive holes from the surface. Hu et al. (2018) indicated that the partial toxicity of TiO_2_ NPs from the photocatalytic activity should be considered because it is difficult to avoid ROS induced by TiO_2_ NPs, despite these substances being almost under dark conditions [32]. Due to the photocatalytic characteristics of TiO_2_ NPs, an abundance of electrons or free radicals were generated via the photocatalytic process to cause toxic effects. In our study, we attempted to prevent the toxic effects of photocatalytic activity as much as possible. For the food additive with TiO_2_ NPs, the photocatalytic toxicity in *C. elegans* after the photocatalytic activity in the environment was not considered in the present study.

This study showed that the exposure of nematodes to either C-TiO_2_ or S-TiO_2_ had no effect on the survival rate or body length of *C. elegans*. However, there was a significant decrease in the reproduction and locomotion function of nematodes, especially after exposure to C-TiO_2_. There was a consistent overexpression of the sod-1, sod-3, ctl-1, ctl-2, cyp35a2, mlt-1, and mlt-2 genes in nematodes that were exposed to 1 mg/L of C-TiO_2_ and 10 mg/L of S-TiO_2_ NPs. These results indicate that nematodes that were exposed to TiO_2_ NPs have impaired reproductive and locomotive function, and the modulation of the expression of these genes is related to the induction of oxidative stress. Although the production of S-TiO_2_ NPs employed environmentally friendly technology, it also generated toxicity toward the nematodes. Compared with S-TiO_2_ NPs, C-TiO_2_ NPs seemed to cause more severe toxicity in *C. elegans*. The findings of the present study show that TiO_2_ NPs, including C-TiO_2_ and S-TiO_2_, had toxic effects on *C. elegans* after the worms were acutely exposed to these nanomaterials, indicating that TiO_2_ NPs as food additives should be associated with safety concerns. Future studies will focus on the specific mechanisms or pathways through which TiO_2_ NPs induce a change in gene expression and affect the reproduction and locomotion function of nematodes.

## 5. Conclusions

This study determined the toxicity of TiO_2_ NPs, including C-TiO_2_ and S-TiO_2_, in *C. elegans*, examining lethality, reproduction, locomotion, longevity, and oxidative stress. TiO_2_ NPs as food additives are banned in the EU, and their toxicity is continuously reviewed by scientists. The results of this study show that while these TiO_2_ NPs did not significantly affect the survival rate or body length of nematodes, they induced significantly negative effects on reproductive function and locomotion behavior. The levels of TiO_2_ NPs (C-TiO_2_ and S-TiO_2_) from ppb to ppm can shorten nematode longevity. There was a concentration-dependent decrease in the brood size, head thrashing, and body bending after exposure to C-TiO_2_ and S-TiO_2_, but C-TiO_2_ had a more pronounced toxic effect. Gene expression analysis shows that there was a consistent overexpression of the key genes associated with oxidative stress and detoxification. These results show that TiO_2_ NPs are toxic and demonstrate their adverse effects. Future studies will determine the specific mechanisms through which TiO_2_ NPs affect nematodes.

## Figures and Tables

**Table 1:** BET surface area, pore volume, pore size, and crystallite size of C-TiO_2_ and S-TiO_2_ ^a^.

Sample	BET Surface Area (m^2^/g)	Pore Volume (cm^3^/g)	Pore Size (nm)	Crystallite Size (nm)
C-TiO_2_	49.3	0.09	8.3	19.2
S-TiO_2_	244.1	0.25	4.8	9.5

^a^ BET: Brunauer–Emmett–Teller; C-TiO_2_: commercially available TiO_2_ NPs; S- TiO_2_: synthetically prepared TiO_2_ NPs.

**Table 2:** Toxicological tests used for the *C. elegans*, with or without TiO_2_ NP treatment.

Toxicological Test	C-TiO_2_ or S-TiO_2_ Concentration (mg/L)	Exposure Period (h)	Number of the Exposed or Untreated Nematodes for Each Dose (n)	Observed Effect on *Caenorhabditis elegans*
Physical characteristics Lethality assay	Control 0.01, 0.1, 1.0, and 10 mg/L	24 h	50	Alive or dead
Growth measurement	Control 0.01, 0.1, 1.0, and 10 mg/L	24 h	30	Body length
Reproductive assay	Control 0.01, 0.1, 1.0, and 10 mg/L	24 h	30	Brood size
Locomotion assay	Control 0.01, 0.1, 1.0, and 10 mg/L	24 h	30	Head thrashing Body bending
Lifespan assay	Control 0.01, 0.1, 1.0, and 10 mg/L	24 h	30	Longevity
Gene expression sod-1, sod-3, ctl-1, and ctl-2	Control 0.01, 0.1, 1.0, and 10 mg/L	24 h	500–1000	Oxidative stress
mlt-1 and mlt-2	Control 0.01, 0.1, 1.0, and 10 mg/L	24 h	500–1000	Stress response or detoxification of heavy metals
cyp35a2	Control 0.01, 0.1, 1.0, and 10 mg/L	24 h	500–1000	Xenobiotic activity and oxidation

**Table 3:** Primer sequences for the *C. elegans* models.

Gene Code	Forward Primer (5′ to 3′)	Reverse Primer (5′ to 3′)
*Caenorhabditis elegans* (*C. elegans*)		
sod-1	TCAGGTCTCCAACGCGATTT	ACCGGGAGTAAGTCCCTTGA
sod-3	CTCCAAGCACACTCTCCCAG	TCCCTTTCGAAACAGCCTCG
ctl-1	GTGTCGTTCATGCCAAGGGAG	TGGATTGCGTCACGAATGAAG
ctl-2	TCCCAGATGGGTACCGTCAT	GGTCCGAAGAGGCAAGTTGA
cyp-35A2	TCGATTTGTGGATGACTGG	AATGGATGCATGACGTTGAA
mtl-1	AGTGCGGAGACAAATGTGAATGC	AGCAGTTCCCTGGTGTTGATGG
mtl-2	TTGTTCCTGCAACACCGGAA	GTTGGCACACTTGCATCCTC
actin-1 ^a^	AGAAGAGCACCCAGTCCTCC	GAAGCGTAGAGGGAGAGGAC

^a^ actin-1 is the control.

## Data Availability

The original contributions are included in the study.
